# A Structure-Function Mechanism for Schizophrenia

**DOI:** 10.3389/fpsyt.2012.00108

**Published:** 2012-12-28

**Authors:** Kunjumon I. Vadakkan

**Affiliations:** ^1^Division of Neurology, Department of Internal Medicine, Faculty of Medicine, University of ManitobaWinnipeg, MB, Canada

**Keywords:** schizophrenia, hallucination, internal sensation, semblance hypothesis, membrane hemi-fusion

## Abstract

The multiple etiologies of schizophrenia prompt us to raise the question: what final common pathway can induce a convincing sense of the reality of the hallucinations in this disease? The observation that artificial stimulation of an intermediate order of neurons of a normal nervous system induces hallucinations indicates that the lateral entry of activity (not resulting from canonical synaptic transmission) at intermediate neuronal orders may provide a mechanism for hallucinations. Meaningful hallucinations can be de-constructed into an organized temporal sequence of internal sensations of associatively learned items that occur in the absence of any external stimuli. We hypothesize that these hallucinations are autonomously generated by the re-activation of pathological non-specific functional LINKs formed between the postsynaptic membranes at certain neuronal orders and are examined as a final common mechanism capable of explaining most of the features of the disease. Reversible and stabilizable hemi-fusion between simultaneously activated adjacent postsynaptic membranes is viewed as one of the normal mechanisms for functional LINK formation and is dependent on lipid membrane composition. Methods of removing the proteins that may traverse the non-specifically hemi-fused membrane segments and attempts to replace the phospholipid side chains to convert the membrane composition to a near-normal state may offer therapeutic opportunities.

## Introduction

Schizophrenia has been viewed as a neuro-developmental disorder (McGorry et al., 2008; Insel, 2010) with stages of pre-symptomatic risk, pre-psychotic prodrome, acute psychosis, and chronic illness, where the last two stages present with delusions, hallucinations, negative symptoms, cognitive alterations, and affective dysregulation (van Os et al., 2010). It is also thought that mental illnesses are determined by their effect on the conscious representational experience (Graham and Stephens, 2007). The features of schizophrenia are known to manifest across all domains of consciousness, namely subjective experience, expression, cognition, affect, behavior, and will (Parnas, 2011), necessitating an explanation for all its different features that stems from alterations of a basic mechanism related to the formation of an internal sense of consciousness. Antipsychotics that reduce delusions and hallucinations (Harvey et al., 2010) do not enhance functional recovery from treatment-refractory cognitive deficits of attention and working memory (Insel, 2010). Due to the difficulties in explaining the patho-physiology of the cognitive defects (Holden, 2003), emphasis has been placed on using knowledge from research work on Alzheimer’s disease (Insel, 2010). Even with all these challenges, there is optimism about the discovery of the neural mechanisms that produce mental experiences (Thagard, 2008).

The nature of the elements contributing to the normal functioning of any complex system may be understood by examining its disease states. However, results from a large number of genomic studies indicate the possibility of a large variety of lesions leading to the prospect that this disease may consist of many different disorders (Kirkpatrick et al., 2001). Varying etiologies of this disorder, including developmental trauma, incidence in minority racial groups, upbringing in an urban environment, and cannabis use, make study of the disorder challenging (van Os et al., 2010). A framework of the disease process is expected to provide an explanation for the positive symptom of hallucinations, the negative symptom of catatonia, cognitive impairment, relief from hallucinations by D2 receptor antagonists, involvement of the disease across all the domains of consciousness, a concordance rate of the disease close to 50% in monozygotic twins, manifestation of the symptoms starting in the teenage period, and schizophrenia-like symptoms in hyper-dopaminergic and *N*-methyl-d-aspartate (NMDA) receptor-blocking conditions. In these contexts, the contrasting but complementary pieces of the disease puzzle may provide clues to understanding the pathology and verifying the operating mechanisms for internal sensations of higher brain functions. An abstract of this work was presented at the Society for Neuroscience annual meeting in 2009 (Vadakkan, 2009).

### From artificial induction of hallucinations to mechanism of internal sensations

Artificial stimulation of neurons within the nervous system can induce different hallucinations (Selimbeyoglu and Parvizi, 2010). Electrical stimulation of the medial temporal lobe can cause vivid experiences of autobiographic memories (Vignal et al., 2007). Hippocampal activation is associated with the actual experience of auditory (Shergill et al., 2000) or visual hallucinations along with activation in higher-order neocortical areas (Oertel et al., 2007). This supports the view that hippocampal hyperactivity might underlie hallucinations and other positive symptoms in schizophrenia (Lodge and Grace, 2007). These findings also indicate that the lateral entry of activity arising autonomously from pathological changes activates intermediate orders of neurons, mis-directing the route of the normal train of internal sensations throughout the nervous system and evoking hallucinations with a compelling sense of reality.

Even though auditory hallucinations are predominant, visual hallucinations are also reported in schizophrenia (David et al., 2011). Since the neuronal orders in the visual pathway are well characterized, we examine the nature of the hallucinations occurring from different pathologies along the visual pathway to understand the nature of their processing. From Table 1, it can be seen that as the location of pathologies causing the lateral entry of activity moves toward higher neuronal orders, the hallucinations become more non-stereotypical and involve a more dynamic sequence of events.

**Table 1:** **List of diseases, nature of visual hallucinations, and possible neuronal orders affected**.

Location	Nature of hallucinations	Neuronal order
Retina and choroid (Siatkowski et al., 1990; Holroyd et al., 1992)	Flickering flashes of light	1
Vitreal detachment (Schmidt et al., 1996)	Brief vertical flashes of light	1
Optic neuritis (Davis et al., 1976)	Spontaneous flashes of light	1
Occipital epilepsy (Panayiotopoulos, 1994)	Brief, stereotyped, fragmentary, and multi-colored lines of simple patterns	∼2–5
Occipito-temporal ictal (Young et al., 1989)	Palinopsia (image recurs immediately after gaze diversion)	∼3–6
Fusiform gyrus activation (Cardoso et al., 2010)	Hallucinations in color	∼3–6
Focal seizures of temporal lobe (Bancaud et al., 1994)	Deja vu, jamais vu	∼4–8
Diffuse Lewy body disease (O’Brien et al., 2005)	Vivid visual hallucinations: colorful and complex involving scenes of people and animals	Many?
Schizophrenia (David et al., 2011)	Convincing sense of reality with lack of stereotypy (contents vary)	Many?

*It is observed that the higher the neuronal orders at which lateral entry of activity occurs, the more well-formed, non-stereotypical, and convincing are the hallucinations produced*.

### Internal sensation of hallucinations and retrieved memories

The observation that both hallucinations and cognitive defects occur with hippocampal pathologies (Vignal et al., 2007) leads to the expectation that the internal sensations of meaningful hallucinations and retrieved memories may share a common mechanism. Hallucinations are viewed as phenomenologically closely related to conscious perception (Llinas and Pare, 1991; Behrendt, 2010) and share some common mechanisms with the conscious recollection of autobiographical memories (Behrendt, 2010). Another view is that hallucinations differ from conscious perceptions only with regard to the extent to which they are constrained by external physical reality; therefore, understanding the mechanism of any one conscious experience is expected to aid in understanding the mechanism of all of them (Uhlhaas and Singer, 2010). Unlike hallucinations caused by most of the pathologies in Table 1 that are generally perceived as unreal by the subjects, the content of hallucinations in schizophrenia is highly meaningful, with a compelling sense of reality (Linn, 1977; Birchwood et al., 2000). These features bring forth three key implications. (a) The convincing nature of the internal sensation of hallucination implies that the operation of the entire system is affected such that there are no separate functioning areas that can identify the hallucinations as unreal. (b) The autonomous nature of hallucinations indicates that at certain neuronal orders, pathologies exist to evoke a continuous flow of these internal sensations. (c) The meaningful and real nature of hallucinations indicates that the system is utilizing the normal operational mechanism of internal sensations of memory retrieval beyond the locations of the pathological changes.

### Examination of internal sensations

Internal sensations vary from simple sensations of the form of an item in an environment to complex sensations of emotions. The present work examines how associated information from the environment is encoded and internal sensations of their memories are formed at a later time point. Since the artificial stimulation of neurons at an intermediate order can induce different hallucinations (Selimbeyoglu and Parvizi, 2010), it can be assumed that pathological conditions inducing the non-specific lateral entry of activity can mis-direct the higher orders of the system to induce an organized temporal sequence of meaningful hallucinations that can explain the symptoms of schizophrenia. The shared elements of the same mechanism should also be able to explain the cognitive defects.

**Figure 1:**
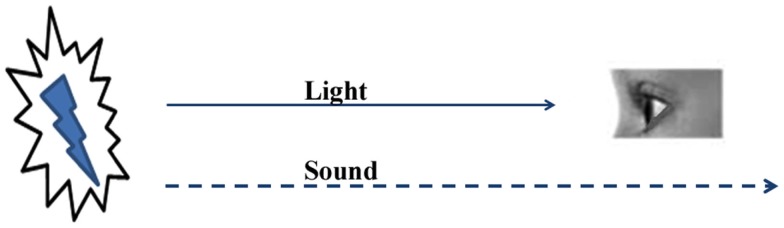
**The nervous system utilizes the early arriving sensations to create the internal sensations of late-arriving or non-arriving sensory stimuli**. The sight of lightning provides the impression that a sound is likely to follow. This property allows the nervous system to anticipate the internal sensations of the remaining sensory stimuli and prepares it to execute motor actions for survival.

The nervous system, with its finite types of sensory systems and finite number of sensory receptors in each system, receives simultaneous sensory inputs to more than one sensory system from an item (“item” means anything that relates to the real world) in the environment. As the distance between the sensory receptors and the item increases, the sensory input traveling at the highest velocity reaches the nervous system faster than the others (Figure 1). The ability to form internal sensations of slowly approaching sensory inputs or inputs that may never reach the nervous system can provide a survival advantage. When an item is very close to an animal, the simultaneous arrival of multiple sensory inputs should make changes in the nervous system so that at a later time when the fastest sensory stimulus arrives alone at the sensory receptors, it creates the semblance of the remaining sensory inputs from the item. A nervous system with this mechanism is expected to build several such operational units during its life-span. At the instance of the arrival of new combinations of sensory stimuli, new combinations of internal sensations can be created, the extent and complexity of which can possibly create complex higher brain functions, including emotions.

### Reducing the internal sensation of memory of an event

Hallucinations induced by the stimulation of an intermediate path by pathological irritation are expected to share some basic features with that of memory retrieval. What kind of reductive approach is required to understand the nature of the internal sensations within the nervous system? An episodic representation of an event is organized into an order of events that unfolds as a mental replay of the event spread over time (Tulving, 2002). Internal sensations of memory for each event can be de-constructed into a series of associative representations of the learned items or events. What basic mechanism at the cellular level can induce changes at the time of associative learning that can be used to evoke the virtual internal sensation of the learned item at the time of memory retrieval? What type of basic unitary structure or its alterations can induce hallucinations of certain sensory stimuli arriving through its preceding neuronal orders and ultimately from the environment, when an intermediate path is activated through lateral entry? A summary of the previously described mechanism (Vadakkan, 2011) is given below.

A novice nervous system consists of synaptically connected neurons, each with varying lengths of dendrites and axonal terminals, arranged in various neuronal orders. On average, a neuron has 2.4–8 × 10^4^ dendritic spines (postsynapses or postsynaptic membranes) on its dendritic tree (Abeles, 1991) where excitatory postsynaptic potentials (EPSPs) can be induced, provided activity arrives at their corresponding presynaptic terminals (presynapses or dendritic spines). The spatial summation of EPSPs from nearly 40 dendritic spines or the temporal summation of EPSPs from fewer than 40 dendritic spines can evoke an action potential at the neuron’s axonal hillock. When the item is close to the animal, different sensory stimuli from the item pass through different neuronal orders and converge at certain neuronal orders. Let us imagine that certain functional LINKs (the letters are capitalized to denote its significance) are formed between two sensory pathways at the locations of their convergence, during associative learning, that can be re-activated by one of the stimuli during memory retrieval. What is an ideal neuronal location for the formation of such a functional LINK between two converging stimulus-induced activities?

**Figure 2:**
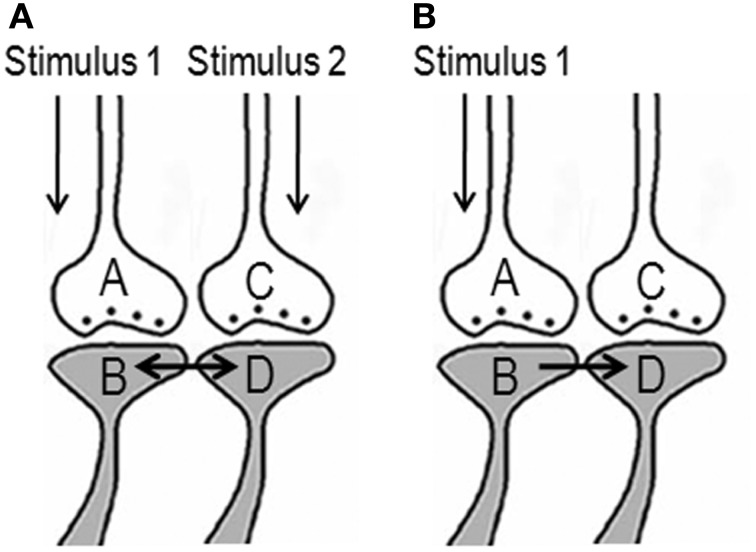
**Potential changes between the postsynapses during associative learning that are later used to induce internal sensation of associatively learned items**. **(A)** The illustration shows functional LINK formation between the two postsynaptic membranes B and D during learning. The functional LINK is transient and its formation is a function of the simultaneous activation of postsynapses B and D. A and C are the corresponding presynapses. **(B)** At a later time, when one the stimuli arrives, the functional LINK is re-activated, resulting in the activation of postsynaptic membrane D inducing the semblance of activity arriving from presynapse C. It is required to maintain the functional LINK in a re-activable state during the interval of time from associative learning to the exposure to one of the stimuli. [Figure was used after modification (Vadakkan, 2011)].

**Figure 3:**
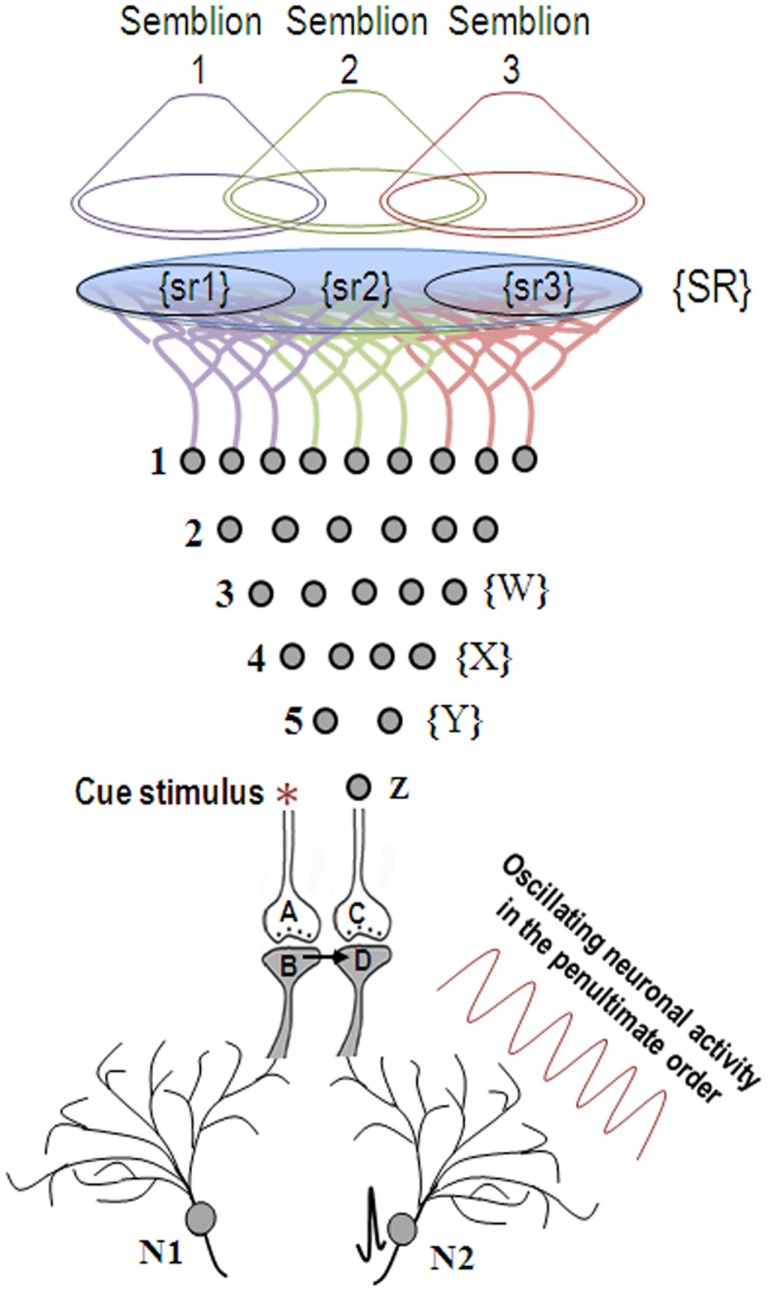
**When the cue stimulus activates synapse A-B, the previously formed functional LINK between postsynapses B and D is re-activated**. Lateral entry of activity activating postsynapse D without receiving activity from presynaptic terminal C evokes a cellular semblance of an action potential reaching from its presynaptic terminal C, which is called synaptic semblance. The nature of the semblance is derived as follows. Postsynapse D experiences the cellular semblance that it is receiving inputs from the set of neurons {Y} that synapse to the neuron Z of its presynapse C. The set of neurons {Y} is activated by the set of neurons {X}, which in turn is activated by the set of neurons {W}. Continuing this extrapolation toward the sensory level identifies a set of sensory receptors {SR}. The nature of the semblance depends on the sensory stimuli that would activate the receptor set {SR}. The stimulation of subsets of sensory receptor sets {sr1}, {sr2}, and {sr3} of the set {SR} may be capable of independently activating neuron Z. The dimensions of hypothetical packets of sensory stimuli capable of activating the sensory receptor subsets {sr1}, {sr2}, and {sr3} are called semblions 1, 2, and 3 respectively and are viewed as the basic building blocks of the virtual internal sensations of memory. The identities of the cue-activated semblances from different postsynapses at each order of neurons (by combination) and the temporal formation of semblances (due to synaptic delay) at different orders of neurons (by permutation) can become integrated to provide the virtual sensation of a sensory stimulus during memory retrieval. Neuronal oscillations and the background sensory inputs result in the summation of EPSPs to only sub-threshold levels in many neurons, short of eliciting action potentials (neuron N2 before the arrival of the cue stimulus). Arrival of the cue stimulus re-activates the functional LINK and activates postsynapse D. When this potential is added to the sub-threshold EPSPs, some neurons (for example, neuron N2) trigger action potential. Thus, the formation of semblances at the functionally LINKed postsynapses may explain the concurrent activation of certain neurons (Reijmers et al., 2007; Gelbard-Sagiv et al., 2008; Tye et al., 2008) by specific cue stimuli during memory retrieval that are likely the neurons that are currently thought to represent memories [Figure was used after modification (Vadakkan, 2011)].

The ideal site should be a sub-cellular location where re-activation of the functional LINK by one of the associatively learned sensory stimuli should be capable of evoking a basic unit of internal sensation of the second stimulus. The structural equivalent of such a LINK should be able to activate the neuronal pathway of the second stimulus at the location of the functional LINK. Moreover, the induced activity at the second pathway should not travel in a retrograde direction. Therefore, the ideal location for the functional LINK is between the postsynaptic membranes at the location of convergence of the two stimuli (Figure 2). Activity reaching the second postsynapse (postsynapse D, Figure 3) through the re-activation of the functional LINK, without any activity arriving from its corresponding presynapse (presynaptic terminal C, Figure 3), induces a normal cellular (physiological) semblance of activity arriving from its presynapse (presynaptic terminal C, Figure 3). The basic units of hypothetical packets of sensory inputs of this physiological semblance at the second postsynapse (postsynapse D in Figure 3) are called semblions (Vadakkan, 2011). This is hypothesized to occur as an intrinsic property of systems in which neurons at certain orders [in the cortex and hippocampus (Buzsaki, 1996)] undergo oscillating activity (derivation given in Figure 3).

**Figure 4:**
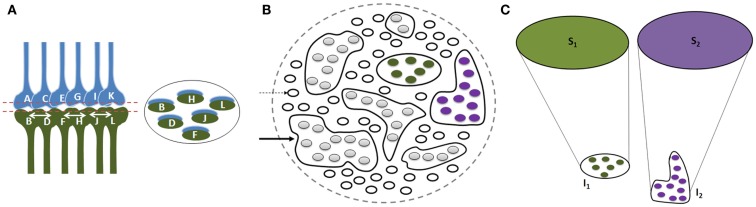
**Illustration showing functional LINK formation between the postsynaptic membranes (postsynapses or dendritic spines)**. **(A)** A group of six synapses are shown close to each other with their postsynapses LINKed by functional LINKs from previous associative learning events. The circular ring shows a cross-section made across the islet of these functional LINKs at the level of the postsynapses. **(B)** Postsynaptic membranes are shown in small circles (broken arrow). Functionally LINKed postsynapses form islets of functionally LINKed postsynapses (solid arrow). **(C)** Semblances S_1_ and S_2_ formed from two islets I_1_ and I_2_ respectively when they are depolarized separately by different cue stimuli during memory retrieval. It is required to maintain the islets of functionally LINKed postsynapses separate and isolated to induce specific semblances by different cue stimuli [Figure was used after modification (Vadakkan, 2011)].

Since physical properties of different items in a given environment share certain common features and since each item in the environment can provide multiple sensory stimuli, different items can stimulate overlapping sets of sensory receptors. Therefore, we expect a large number of inter-postsynaptic functional LINKs to be shared; their number increases as the nervous system is exposed to an increasing number of items during different associative learning events in life. Multiple instances of associative learning lead to the formation of groups of functionally LINKed postsynapses that are called islets (Figure 4). During memory retrieval, this enables the spread of activity within the islet to form semblances of associatively learned related sensory inputs, presenting the common physical properties characteristic of an environment. The net semblances from all the postsynapses at various orders of neurons finally determine the nature of the internal sensations. Repeated re-activation of the commonly used functional LINKs may induce cellular changes to maintain them as near-structural LINKs.

### Failure to keep the islets separate can induce autonomous hallucinations

**Figure 5:**
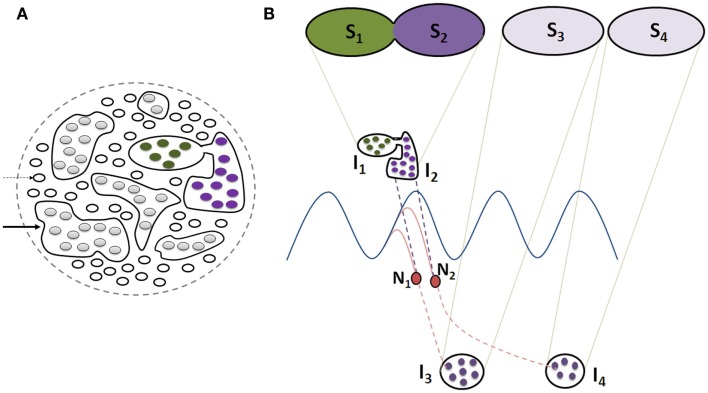
**Schematic representation of the effect of non-specific LINKs between the islets of functionally LINKed postsynapses that can lead to cognitive dysfunction, changes in oscillating waveforms, and result in meaningful hallucinations**. **(A)** Cross-section through large number of postsynapses assuming that they are in the same plane. Thin arrow: single postsynapse; Thick arrow: islet of LINKed postsynapses. Note that two of the islets are interconnected in comparison with those shown in Figure 4B. **(B)** This figure is modified from Figure 4C. Cross-section through two islets of LINKed postsynapses I_1_ and I_2_ (also shown in Figure 4C) that are non-specifically LINKed. This results in the formation of a combination of semblances S_1_ and S_2_, changing the conformation of semblances of retrieved memories (as shown in Figure 4C). Note that the neurons N_1_ and N_2_ that otherwise receive sub-threshold stimuli now start receiving EPSPs from the islet I_1_. This leads to the activation of the neurons N1 and N2, resulting in the activation of islets of functionally LINKed postsynapses I_3_ and I_4_, eliciting semblances (S_3_ and S_4_) of previously associatively learned items or events, producing meaningful hallucinations. Note that background oscillatory neuronal activity was proposed to result in semblance formation from a large number of functionally LINKed postsynapses, resulting in C-semblance responsible for consciousness (Vadakkan, 2010). Non-specific LINK induced activation of large number of neurons similar to N1 and N2 can lead to a change in oscillatory neuronal activity (not shown) changing the conformation of C-semblance (not shown) and produce alterations in conscious perception of items or events.

Keeping the islets of functionally LINKed postsynapses separate from each other is crucial for maintaining the specificity of formed semblances in response to a cue stimulus. Therefore, we can reasonably expect that the formation of any non-specific inter-postsynaptic hemi-fusion may lead to the formation of non-specific semblances during memory retrieval. If this hemi-fusion occurs between single postsynapses, then the net non-specificity of semblances from all the neuronal orders will be comparatively small. However, non-specific LINKs between the postsynapses that are part of large islets of functionally LINKed postsynapses can produce a large reduction in the specificity of semblances at the level of that neuronal order resulting in cognitive impairments (Figure 5). Correlations between reduced volume (Heckers, 2001) of the hippocampus and auditory hallucinations in schizophrenia indicate the possibility for pathologically introduced non-specific functional LINKs between the postsynapses that can become the triggering point for an autonomous and mis-directed train of activity at higher neuronal orders. Surgical removal of the hippocampus was considered as a treatment option to reduce delusions in schizophrenia (Mikell et al., 2009), supporting the view that hippocampal pathologies are capable of triggering autonomic mis-regulated activities.

Non-specific inter-postsynaptic LINKs occurring at the penultimate neuronal order from an oscillating neuronal area can lead to different consequences. (a) Normally, oscillatory neuronal activity is expected to produce sub-threshold activation of those neurons at the higher orders that receive fewer EPSPs than required for spatial or temporal summation capable of inducing an action potential. When additional EPSPs reach these sub-threshold activated neurons, it will lead to their autonomic activation and result in the transmission of activity to higher neuronal orders, activating many previously formed specific functional LINKs and inducing meaningful hallucinations (Figure 5). (b) The non-specific inter-postsynaptic LINKs occurring at the penultimate neuronal order from the oscillating neuronal orders can also lead to changes in oscillatory neuronal activity (Figure 5) and are expected to be responsible for changes in consciousness (Vadakkan, 2010). Such changes in neuronal oscillations in schizophrenia have been extensively reviewed (Uhlhaas and Singer, 2010). Even though inter-postsynaptic changes have not been examined, a large number of studies reporting dendritic spine changes along with gray matter changes in schizophrenia (Bennett, 2011) increase the likelihood of non-specific inter-postsynaptic functional LINKs.

In summary, (a) non-specific inter-postsynaptic functional LINK formation can induce a universal effect at the neuronal orders above the oscillating orders such that no separate functioning areas exist that can identify the hallucinations as unreal. (b) In the presence of the continuous arrival of activity through the pathological non-specific inter-postsynaptic mis-LINKs, the sub-threshold-activated neurons maintained by oscillatory neuronal activity start firing, re-activating the normal inter-postsynaptic functional LINKs at higher neuronal orders so that the hallucinations become autonomous in nature. (c) The convincing sense of reality of the hallucinations indicates that beyond the origin of autonomous activation of non-specific functional mis-LINKs, the system is operating by using the normal mechanism of formation of semblances for memory retrieval, using previously formed inter-postsynaptic functional LINKs from associative learning events; but their formation occurs in a wrong context, inducing hallucinations. (d) The hallucinations are similar to the perception of sensory stimuli from the environment due to the inter-postsynaptic mis-LINKs at the early neuronal orders in the auditory cortex (and not merely the type of internal sensations occurring at the time of memory retrieval by integration of semblances from specific inter-LINKed postsynapses at higher neuronal orders at which the cue stimulus and item whose memories are to be retrieved exist).

### Delusions operate through a similar mechanism

A delusion is a belief held with a very strong conviction by the subject, even in the presence of a counter-argument provided by a second person. This indicates that there is a compelling internal sensation experienced by the subject who is perceiving the delusions and that the subject lacks an operational mechanism within the remaining nervous system that identifies the internal sensation of delusions as unreal. The formation of these delusions can be explained as resulting from their autonomous activations from pathological non-specific inter-postsynaptic functional LINKs. Since the latter leads to the activation of a non-specific set of otherwise normal inter-postsynaptic functional LINKs at higher neuronal orders in the entire nervous system, the subject autonomously continues to perceive items or events (a) that were not associatively learned in the past and (b) that were not associatively learned from the environment from which their nervous systems continue to make associations.

### Reversible and stabilizable membrane hemi-fusion as a mechanism for functional LINKs

**Figure 6:**
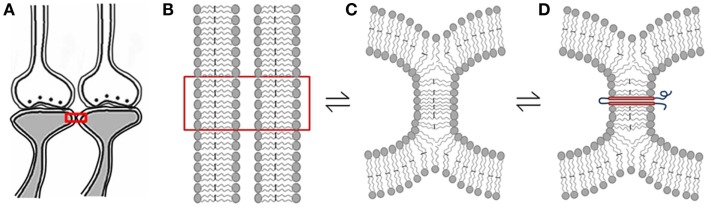
**Schematic diagrams showing hemi-fusion between the lipid bilayers of the postsynaptic membranes as one of the possible mechanisms of formation of functional LINKs between the postsynaptic membranes (postsynapses or dendritic spines)**. **(A)** Closely located postsynaptic membranes that are simultaneously activated during associative learning. **(B)** The boxed region in figure **(A)** is enlarged to show adjacently located postsynaptic membrane lipid bilayers. **(C)** Cellular changes during associative learning can lead to postsynaptic membrane hemi-fusion. If not re-activated, hemi-fused membranes reverse back to independent membranes, making the hemi-fused state a transient one. **(D)** A protein molecule is shown traversing the hemi-fused membrane making the hemi-fused area temporarily stable, depending on the life-span of the inserted trans-membrane proteins or their ability to shift away from the hemi-fused areas by lateral movement. The orientation of N and C terminal ends of these expected trans-membrane proteins are to be examined. Note that the reversible nature of the membrane hemi-fusion is marked by reversible signs between the figures **(B–D)**.

In many locations in the nervous system, the postsynaptic membranes belonging to different neurons are apposed to each other with minimal visible extracellular matrix volume, especially in locations where sensory inputs converge; for example, the hippocampi. One of the feasible mechanisms for the generation of functional LINKs between the postsynaptic membranes is the formation of reversible membrane hemi-fusion. A large number of studies have shown reversible hemi-fusion occurring between biological membranes (Melikyan and Chernomordik, 1997; Chernomordik and Kozlov, 2008; Kozlov et al., 2010). It is reasonable to expect reversible membrane hemi-fusion between the postsynapses during associative learning that can later be re-activated during memory retrieval, resulting in the spread of activity across the hemi-fused membranes to the second postsynapse (Figure 6). After one associative learning event, if the hemi-fused membranes are not reused or re-activated, they will reverse back to independent membranes. If hemi-fused membrane segments are traversed by proteins and are not reused or re-activated, they can reverse back to independent membranes once the life of the trans-membrane proteins is over. If the hypothesis is true, the formation of the non-specific membrane hemi-fusion should be responsible for the induction of hallucinations (note: hereafter, inter-postsynaptic functional LINKs and hemi-fused postsynaptic membranes are used interchangeably).

### Supporting evidence for postsynaptic membrane hemi-fusion

Since internal sensations of hallucinations and retrieved memories are expected to share a common mechanism of operation, we expect inter-postsynaptic functional LINKs to be observed as inter-postsynaptic membrane hemi-fusions. Since extensive research work has already been done in the field of memory, both functional and structural evidence were examined from the literature.

#### Functional evidence

From the studies of patient H.M. (Scoville and Milner, 1957), it was found that H.M. was unable to make any motor expression indicative of experiencing the internal sensations of retrieval of memories of information acquired during a certain period of time prior to the surgical removal of his hippocampi. Following this study, experiments were carried out in rodent hippocampi to study memory. Using hippocampal slices, it was found that if a brief repetitive stimulation is applied initially at the axonal regions of the neurons of CA3 layer of neurons (Schaffer collaterals), then the application of a regular stimulus at the same location is sufficient to induce a potentiated effect at the CA3-CA1 synapses as observed by recording from the CA1 region and is called long-term potentiation (LTP; Bliss and Lomo, 1973). A large number of correlations between behavioral motor outputs, indicative of memory retrieval, and LTP were reported (Morris et al., 2003; Whitlock et al., 2006). Based on the hypothesis that semblance formation is responsible for the internal sensation of memories, inter-postsynaptic membranes are expected to become hemi-fused during both associative learning and LTP induction. Experimental evidence for membrane hemi-fusion during associative learning may provide indirect evidence for the formation of non-specific inter-postsynaptic hemi-fusions in schizophrenia.

In agreement with the above, postsynaptic membrane fusion was observed during the induction of LTP by introducing exogenous synaptosomal-associated protein (SNAP) into the postsynapses through the cell body of the neuron (Lledo et al., 1998). Since this postsynaptic fusion occluded further LTP induction and prior LTP induction independently occluded SNAP-induced LTP induction, is was reported that postsynaptic membrane fusion takes place during LTP (Lledo et al., 1998). However, it is not known where such membrane fusions take place at the postsynaptic membrane. Since multiple axons are activated simultaneously during LTP induction and since many postsynapses of the synapses at their axonal terminals (presynapses) are likely apposed to each other, it is reasonable to argue that inter-postsynaptic membrane hemi-fusion occur during LTP induction. Combined with the observed correlations between behavioral motor features of memory and LTP, this provides indirect evidence for the formation of inter-postsynaptic membrane hemi-fusion during associative learning.

#### Structural evidence

To seek structural evidence for postsynaptic membrane fusion, electron microscopic (EM) images of the synapses from previous studies were examined. By the time rodents reach adulthood, it is expected that they have experienced a large number of associative learning events. Therefore, hemi-fused inter-postsynaptic membranes at various locations in the nervous system are expected to be found. Even though limited resolution sometimes reduces the view of the membrane lipid bilayers to only single layers, overlapping of the adjacent postsynaptic membranes can be observed from the results of different studies (Burette et al., 2012) and (Harris and Stevens, 1989; He et al., 1998; Sirvanci et al., 2005) indicating the possible presence of inter-postsynaptic membrane hemi-fusion at those locations.

### Phospholipids and membrane hemi-fusion

**Figure 7:**
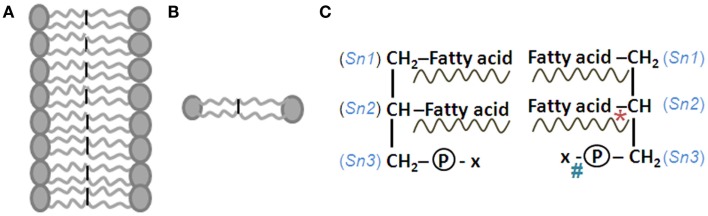
**Structure of lipid membrane and its components**. **(A)** Cell membrane lipid bilayers where the constituent phospholipid molecules have their hydrophobic ends tuned toward the interior and hydrophilic ends facing outside. **(B)** Two phospholipid molecules facing each other. **(C)** Structures of these phospholipid molecules are shown as mirror images. Sn, side chain number. At the third position, x stands for choline, ethanolamine, inositol, or serine. *Location of action of phospholipase A2 enzyme. #Location of action of phospholipase D. Phospholipids are named for the presence of phosphate group. Their main structural stem is a molecule of glycerol, a 3-carbon alcohol. The fatty acid side chains may be saturated (contains no double bonds and a name that ends in -anoic acid) or unsaturated (contains double bonds and a name that ends in -enoic acid). Unsaturated fatty acid contributes to the fluidity of the membranes. Double bonds can be introduced at the Δ^4^, Δ^5^, Δ^6^, and Δ^9^ positions, but not beyond the Δ^9^ position (Δ denotes the position of double bonds from the carboxylic acid terminal; ω denotes the position of double bonds from the methyl terminal end). Usually the fatty acid chains with an even number of carbon atoms attach to the first (Sn-1; saturated fatty acids such as palmitic or stearic acid) and second (Sn-2; unsaturated fatty acids such as oleic or arachidonic acid) carbons of the glycerol molecule. Linoleic acid and alpha-linolenic acid are the only essential fatty acids (EFAs) that must be obtained from the diet. All other fatty acids can be synthesized within the body. Alternate steps of elongation (by the enzyme elongase) and desaturation (by Δ desaturase enzyme) enable the synthesis of polyunsaturated fatty acids (PUFAs) from EFAs. The Sn-3 position hydroxyl group bonds with phosphoric acid to form phosphatidic acid that in turn bonds with an alcohol such as serine, ethanolamine, choline, inositol, and ethanolamine. When the alcohol is sphingosine, the molecule is called sphingo-phospholipid. Fatty acids are transported in the blood either by binding to albumin or in the form of triglycerides associated with lipoproteins. From lipoprotein triglycerides, the fatty acids are released by the action of lipoprotein lipase in the endothelial cells of the capillaries (Spector and Yorek, 1985; Ben-Zeev et al., 1990). Lipoprotein lipase levels are found higher in the hippocampus and neocortex (Ben-Zeev et al., 1990). Choline-containing phospholipids are preferentially located in the outer layer and phosphatidyl serine and phosphatidyl ethanolamine-containing phospholipids are located in the inner layer of the membrane bilayer (Murray et al., 2006). The enzyme flippases transfer phospholipids to different layers. Membranes also have cholesterol, cholesterol esters, and protein molecules embedded within them.

The attachment of different molecules at both the *Sn-2* position and to the phosphoric acid at the *Sn-3* position is determined by the substrate availability as well as sequential activities of enzyme pairs at these locations; both of these conditions are essential for switching the side chain fatty acids (Figure 7). The exchange between fatty acids at the *Sn-2* position is carried out by the enzymes phospholipase A2 (PLA2; removes fatty acid) and acyl transferase (adds fatty acid; Murray et al., 2006) and the exchange between phosphatidyl choline (PC) and phosphatidyl ethanolamine (PE) attached to the phosphatidic acid at the *Sn-3* position is carried out by sequential actions of phospholipase D and acyl transferase enzymes. There is preferential distribution of arachidonic acid (AA) and docosa-hexaenoic acid (DHA), both derived from essential fatty acids (EFA), in the *Sn-2* position in neuronal cells (Sugiura et al., 2009). It was shown that monolayers formed by cone-shaped PE and diacyl glycerol (DAG) bulge in the direction of the non-polar hydrocarbon chains, whereas the inverted cone-shaped lysophosphatidyl choline (LPC) bulges in the opposite direction (Chernomordik and Kozlov, 2003). This shows that lipid bilayer composition can determine the bulging of the apposed membranes and possibly lead to hemi-fusion. These findings reinforce the argument that the nature of side chains of phospholipids can determine hemi-fusion between postsynaptic membranes, either in response to their simultaneous activation during associative learning or non-specifically, in the latter case leading to the patho-physiology in schizophrenia.

### Observed correlations with lipid metabolism

An examination of studies of schizophrenia shows supportive evidence for possible errors in hemi-fusion between the postsynaptic membranes.

#### Membrane phospholipid change

Essential fatty acid levels were found to be lower in the plasma and red cell membranes of schizophrenic patients (Horrobin et al., 1989; Kaiya et al., 1991). Similar findings in the plasma were found in twins concordant for schizophrenia (Bates et al., 1991). Furthermore, it was reported that circulating levels of PLA2 activity are increased in the blood of schizophrenic patients (Gattaz et al., 1990; Gattaz and Brunner, 1996; Ong et al., 2010). Genetic abnormalities were also observed in the vicinity of the *PLA2* gene (Hudson et al., 1996). There are reports of an increased rate of loss of essential polyunsaturated fatty acids (PUFAs) from the *Sn-2* position of phospholipids in schizophrenia (Glen et al., 1994; Horrobin et al., 1994; Peet et al., 1994). It has also been found that levels of some of the essential PUFAs (AA and DHA) are reduced in their red cell membrane phospholipids (Glen et al., 1994; Peet et al., 1994). Similarly, different tissues were found to be deficient in essential PUFAs in schizophrenic patients (Yao et al., 1994a,b, 2002; Doris et al., 1998; Assies et al., 2001; Khan et al., 2002; Peet and Horrobin, 2002; Arvindakshan et al., 2003; Reddy et al., 2004). Decreased essential PUFA levels were also shown to be correlated with impaired cognitive functioning in schizophrenic patients (Sumiyoshi et al., 2008). Based on different correlational findings, abnormal phospholipids in the membranes were hypothesized in the patho-physiology of schizophrenia (Horrobin et al., 1994; Gattaz and Brunner, 1996; Horrobin, 1998). Later, a large number of investigations showed support for this view (Glen et al., 1994; Fenton et al., 2000; Maekawa et al., 2011). Furthermore, magnetic resonance imaging (MRI) studies have found an increased rate of phospholipid breakdown in the brain of un-medicated schizophrenics (Pettegrew et al., 1991; Williamson et al., 1996; Bentsen et al., 2012). The effects of reduced PUFAs in the phospholipids either by reduced EFA in the diet or by defects in the enzymes taking part in side chain fatty acid replacements or a combination of the two is likely to predispose the postsynaptic membranes to undergo non-specific hemi-fusions.

#### Essential fatty acids in the diet

A recent randomized, placebo-controlled study showed a significant effect of long-chain omega-3 fatty acids in preventing the development of psychotic disorders in adolescents presenting with the known prodrome for the disease (Amminger et al., 2010). In one study, the essential PUFA concentrations were positively related with an improvement of positive symptoms (Sumiyoshi et al., 2011). Another study carried out by treating schizophrenia with EFAs, specifically γ-linolenic acid or dihomogamma-linolenic acid, along with factors for EFA metabolism such as zinc, vitamins C, B6, and niacin, showed substantial improvement in symptoms along with an elevation in red cell membrane EFAs (Vaddadi, 1992). Furthermore, a blood marker of EFA deficiency, the triene: tetraene ratio (Lee et al., 1993) was found decreased in schizophrenics (Assies et al., 2001). These findings raise the possibility that if the limiting EFAs are not obtained through food, fatty acids in the *Sn-2* position may get replaced (Figure 5) with saturated fatty acids, especially in the setting of increased PLA2 enzyme. A deficiency of EFA in the diet can lead to the replacement of their derivatives with non-essential polyenoic acids at the *Sn*-*2* position in phospholipids (Murray et al., 2006). The fatty acid compositional change in the phospholipids of the membranes may lead to non-specific hemi-fusion between the postsynapses, a possible stable low-energy state. It is possible that through the replacement of fatty acids both at *Sn-2* and *Sn-3* positions, EFA supplementation may lead to the incorporation of long-chain PUFAs and reverse the pathology in schizophrenia. Even though treatment with EFA may not reverse previously hemi-fused membranes that are traversed by trans-membrane proteins, it may halt the formation of further non-specific inter-postsynaptic membrane hemi-fusions (Amminger et al., 2010; Sumiyoshi et al., 2011).

#### Linkage analysis

A large number of linkage analysis studies have shown putative candidate genes whose protein products are associated with lipid metabolism in schizophrenic patients and their family members (Moises et al., 1995; Pulver et al., 1995; Straub et al., 1997; Blouin et al., 1998; Hovatta et al., 1998; Shaw et al., 1998; Barnes et al., 1999; Brzustowicz et al., 1999; Gurling et al., 1999; Mors and Ewald, 1999; Riley and Williamson, 2000; Table 2). Among these, micro-deletion of chromosome 22q11 results in the development of a form of schizophrenia among 30% of children with this deletion (Karayiorgou et al., 2010). These multiple associations may indicate their role in maintaining normal phospholipid composition; defects in these chromosomes in combination with dietary deficiencies may initiate the formation of non-specific inter-postsynaptic hemi-fusions.

**Table 2:** **Chromosome locations of some of the genes involved in lipid metabolism that are associated with schizophrenia**.

Locations of mutations in schizophrenic families	Proteins involved in the metabolism of phospholipid and fatty acids	Chromosome locations of their genes
2p25-p21 (Moises et al., 1995)	Apolipoprotein B	2p24
11q22-q23 (Riley and Williamson, 2000)	Apolipoprotein A-IV	11q23
1q22-q23 (Shaw et al., 1998)	Apolipoprotein A-II	1q21–1q23
16q22 (Shaw et al., 1998)	Lecithin: cholesterol acyl transferase	16q22.1
8p21-p23 (Pulver et al., 1995; Straub et al., 1997; Blouin et al., 1998; Brzustowicz et al., 1999; Gurling et al., 1999; Mors and Ewald, 1999)	Lipoprotein lipase	8p21–22
7q11 (Blouin et al., 1998)	Fatty acid transport protein	7q11.2
9q34.3 (Riley and Williamson, 2000)	Carnitine acetyl transferase	9q34.1
11q14-24 (Gurling et al., 1999)	Carnitine palmitoyl transferase-I	11q13.1–13.5
2q21 (Barnes et al., 1999)	Acyl coenzyme A binding protein	2q12–q21
9p23-p21 (Moises et al., 1995)	Phospholipase A2 activating protein	9p21
9q32 (Barnes et al., 1999)	Cyclooxygenase-1	9q32–33.3
20p12 (Moises et al., 1995)	Phospholipase C, Beta 4	20p12
20q13 (Barnes et al., 1999)	Phospholipase C, Gamma 1	20q12–q13.1
22q11-q13 (Pulver et al., 1995, 22q (Hovatta et al., 1998)	Phospholipase A2, Group VI	22q13.1

*Chromosome locations of some of the genes whose proteins are involved in various stages of the fatty acid transport, phospholipid side chain replacements, and inter-conversion of different lipoproteins are shown*.

## Explanation for the Remaining Features of Schizophrenia

### Cognitive impairment

It is thought that the disability of schizophrenia results more from under-recognized and treatment-resistant cognitive deficits than from more visible treatment-sensitive positive symptoms (Harvey et al., 2010). Data from the Dunedin birth cohort studies (Walsh et al., 2008) indicate that intelligence quotient (IQ) is reduced early and persistently in children who eventually develop schizophrenia (Li et al., 2007). Furthermore, it has been found that reduced working memory and poor cognitive control are the core features that lead to long-term morbidity and poor functional outcomes in schizophrenia (Hyman and Fenton, 2003). Abnormal functioning of the left temporal lobe is observed in schizophrenia (Shenton et al., 1992). Two meta-analyses (Lawrie and Abukmeil, 1998; Nelson et al., 1998) and one large cohort study (Gothelf et al., 2000) have shown that the hippocampus is smaller in schizophrenic patients than controls by approximately 5%. Imaging studies have shown hippocampal volume reduction and abnormal levels of hippocampal activity at rest and during the motor outputs associated with memory retrieval (Heckers, 2001). Pathological changes at the prefrontal cortex (Kraepelin, 1919) and hippocampus (Bogerts et al., 1985; Harrison, 2004) were reported in schizophrenia.

#### Working memory

It is thought that a reduction in working memory may be the best predictor of the psychotic phase of schizophrenia (Coyle, 2006). Based on the neuro-developmental disorder view of schizophrenia, cognitive impairment starts to manifest during the stage two of the disease (McGorry et al., 2008; Insel, 2010). Based on the present work, working memory uses the maximum number of specific newly hemi-fused postsynaptic membranes to induce a maximum of specific semblances for memory. Non-specific postsynaptic membrane hemi-fusions increase non-specific semblances at these locations. Since functional LINKs occurring at the penultimate orders from the oscillating neuronal orders can activate sub-threshold-activated neurons, the resulting non-specific semblances can reduce working memory.

#### NMDA receptor antagonism leads to schizophrenia-like symptoms

*N*-methyl-d-aspartate receptor antibody encephalitis induced by auto-antibodies to NR1/NR2B heteromers of the NMDA receptors manifests as severe memory defects and schizophrenia-like symptoms of hallucinations (Coyle, 2006; Dalmau et al., 2008). Furthermore, a significant reduction in kynurenine 3-mono-oxygenase enzyme that metabolizes kynurenic acid, an antagonist of NMDA, is reported in schizophrenia patients (Wonodi et al., 2011). Based on the present work, an acute reduction in NMDA activity attenuates neurotransmission at certain excitatory synapses, resulting in a reduced ability to re-activate functional LINKs, and alters neuronal oscillations, leading to cognitive defects and schizophrenia-like symptoms.

#### Dopamine, memory, and LTP

Based on the present hypothesis, the finding that dopamine promotes motivation-induced learning (Wang et al., 2004) can be attributed to dopamine’s role in functional LINK formation. Therefore, dopamine antagonists are likely to exhibit an opposite effect: reducing cognitive abilities. In an earlier section, we explained how LTP induction may result in the formation of a large number of inter-postsynaptic functional LINKs. Since dopamine is implicated in long-term memory storage (Rossato et al., 2009) and has been found to be necessary for late-LTP at the CA3-CA1 synapses of the hippocampi (Matthies et al., 1997; Swanson-Park et al., 1999; Yang et al., 2002; O’Carroll and Morris, 2004), it is possible that dopamine augments or stabilizes the inter-postsynaptic functional LINKs.

### Effect of DOPA receptor-blocking antipsychotics on hallucinations

Based on the present work, factors that promote the formation of inter-postsynaptic functional LINKs are likely to augment learning; those that inhibit are likely to attenuate learning. The effect of dopamine on both motivation-induced learning (Wang et al., 2004) and on the persistence of long-term memory storage (Rossato et al., 2009) suggests the possibility that dopamine promotes and maintains inter-postsynaptic functional LINKs. We can extend this view to argue that hyper-dopaminergic conditions may lead to the formation of additional non-specific functional LINKs during spontaneous activity of dopaminergic neurons in the ventral tegmental area, which was shown to produce symptoms of psychosis (Liddle et al., 2000; Lodge and Grace, 2007). This may also explain why schizophrenia is viewed as a dopamine disorder based on the psychosis-inducing effects of dopamine-releasing drugs such as amphetamine, and the antipsychotic efficacy of drugs that block the dopamine D2 receptors (Okubo et al., 1997). Supporting evidence was also observed in animal experiments. D2 receptor over-expressing transgenic mice (Castner et al., 2000) that were bred to test the dopamine hypothesis (Sedvall and Farde, 1995; Okubo et al., 1997) showed substantial behavioral changes related to prefrontal cortical function. Dopamine’s role in modulating neurotransmission in the prefrontal cortex has also been demonstrated (Burmeister et al., 2008; Stefansson et al., 2008). Furthermore, micro-deletion of the catechol *o*-methyl transferase (COMT) gene causes deficiency of its protein, leading to reduced catabolism of dopamine and further leading to the latter’s accumulation and resulting in an increased susceptibility to schizophrenia (Egan et al., 2001).

In separate studies, both reward and aversive stimuli were shown to cause the bursting of dopaminergic neurons (Ljungberg et al., 1992; Schultz, 2007; Bromberg-Martin et al., 2010), releasing dopamine at their nerve terminals. Electro-physiological recordings have demonstrated the activation of the human substantia nigra or ventral tegmental areas by associative reward stimuli (Zaghloul et al., 2009). A similar observation was made for one-trial encoding of the location of the escape platform in a water maze (O’Carroll et al., 2006). Furthermore, intra-hippocampal infusion of the D1/D5 dopamine antagonist just before encoding was shown to cause impaired behavioral motor outputs (Bethus et al., 2010). Further support comes from reports that amphiphilic psychotropic drugs stimulate cellular lipid biosynthesis (Vik-Mo et al., 2009) through the activation of the sterol regulatory element-binding protein transcription factors, regulating the expression of many genes during fatty acid synthesis. Thus, a mechanistic explanation is possible to define the role of dopamine in inducing and maintaining specific inter-postsynaptic membrane hemi-fusion.

### Relation with consciousness

Fundamental features of schizophrenia have been observed to manifest across all the domains of consciousness: subjective experience, expression, cognition, affect, behavior, and will (Parnas, 2011). It is viewed that what defines a mental illness is its effect on the conscious representational experience (Anscombe, 1987; Graham and Stephens, 2007). Moreover, schizophrenia is thought to involve a profound alteration in the structures (frameworks) of subjectivity (consciousness), and to manifest in self-relation and in the relation to the world (Urfer-Parnas et al., 2010). Furthermore, it is thought that schizophrenic patients lack control over mental processes so that when emotions and intuitions are not brought into focus, the individual loses his or her sense of self (Krabbendam and van Os, 2005). It is reasonable to argue that a mechanism that can explain the formation of internal sensations should also be able to explain how this basic mechanism can be expanded to involve different domains of consciousness. In this regard, baseline neuronal oscillations in the hippocampus and cortex and background sensory inputs are postulated to induce re-activations of a large number of functional LINKs, inducing a net non-specific semblance called C-semblance, which is responsible for normal consciousness (Vadakkan, 2010). According to the present framework, when non-specific hemi-fusion between the postsynaptic membranes takes place at certain neuronal orders, the alterations in the oscillatory waveforms at the higher neuronal orders change the conformation of C-semblance. The impairment of stimulus-induced gamma synchronization across neocortical networks (Schiller, 1992), and an impaired response to Gestalt (Spencer et al., 2003), and auditory and sensory stimuli (Green et al., 1999; Kwon et al., 1999) were reported in schizophrenic patients, which is in agreement with the present framework. It is possible that the non-specific functional LINKs distort the oscillatory waveforms and the resulting C-semblances altering the conscious state.

### Negative symptoms

Non-specific inter-postsynaptic functional LINKs can lead to autonomous activity that can stimulate non-specific sets of neurons in the motor cortex, causing non-purposeful motor outflow, and resulting in the symptoms of catatonia. Trains of unmatched semblances may explain disorganized hallucinations. As the number of non-specific functional LINKs increases, it will result in bizarre non-meaningful hallucinations.

### Explanation for the observed concordance rates

Even though studies of generations of families and twins have demonstrated the high heritability of schizophrenia (Purcell et al., 2009; Stefansson et al., 2009; Ettinger et al., 2012), the concordance rate in monozygotic twins affected with the disease is found to be roughly 45–50% (Kallmann, 1938; Kety, 1987; McGuffin and Gottesman, 1999). If schizophrenia is caused entirely by genetic abnormalities, the concordance rate in monozygotic twins who have almost identical genes is expected to be nearly 100%. The concordance rate in dizygotic twins is nearly 15%, which is about the same as for other siblings (Kallmann, 1938). The rate of schizophrenia was found to be higher among the biological relatives of adopted children who developed schizophrenia than among adopted children who were normal (Heston, 1970; Rosenthal et al., 1971), indicating that related protein products can undergo certain processes that predispose them to the disease process.

The concordance rate of nearly 50% among monozygotic twins may be explained as follows. The present work has shown how the replacement of PUFAs with saturated fatty acids at the *Sn-2* position of the phospholipids may facilitate non-specific hemi-fusion between the postsynaptic membranes. Multiple genetic loci code for proteins in the lipid metabolic pathways, some of which are enzymes (Table 2); these loci are implicated in the etiology of schizophrenia. Autosomal recessive inheritance of enzymes is expected to result in heterozygotes with reduced expression of the protein from a single functional allele of the gene. These heterozygotes remain symptom-free, provided they receive sufficient EFA in the diet. However, a dietary deficiency of EFA can lead to reduced incorporation of PUFA at the *Sn-2* position, which in turn may produce non-specific membrane hemi-fusion. Since the probability of PUFAs being made and incorporated into the membrane phospholipids ranges from 0 to 100, an average value of 50% may explain the nearly 50% concordance rate among monozygotic twins.

Another mechanism also needs mention. Schizophrenic patients have shown global hypo-methylation within the DNA, which is known to reverse with neuroleptic medications (Melas et al., 2012). Reduced methylation of DNA increases gene expression, possibly to compensate for the reduced expression of certain proteins. While this may be beneficial for maximizing expression from single alleles in heterozygotes, it can lead to the over-expression of certain other proteins that may adversely affect the disease process. For example, PLA2 over-expression in schizophrenic patients (Gattaz et al., 1990; Gattaz and Brunner, 1996) removes DHA and AA from *Sn-2* position of phospholipids. In the case of reduced dietary EFA, it can lead to reduced synthesis of PUFA, further leading to the incorporation of saturated fatty acids at the *Sn-2* position of the phospholipids, which can predispose the membranes to non-specific hemi-fusion.

### Initial manifestation in teenage groups

Psychosis emerges in late adolescence or early adulthood, with peak incidence between ages 18 and 25, when the prefrontal cortex continues its development. Longitudinal neuro-imaging studies have demonstrated changes in gray matter density until the mid-twenties, with the prefrontal cortex being the last to mature (Rakic et al., 1986; Lewis and Gonzalez-Burgos, 2008; Hashimoto et al., 2009). Dopaminergic innervation of the prefrontal cortex increases markedly during adolescence (Woodberry et al., 2008). A report from a 45 year follow up of a Copenhagen birth cohort demonstrates that adults with schizophrenia have a history of delayed developmental milestones in the first year followed by delayed maturation (Reichenberg et al., 2010). Even though a disruption of the genes that are either associated (Niwa et al., 2010) or not associated (Tan et al., 2007; Colantuoni et al., 2008; Nakata et al., 2009) with neuronal development is implicated in schizophrenia, the role of this disruption in disease development is not known. From a structural point of view, the “mis-wiring” hypothesis of schizophrenia is based on the aberrant synaptic pruning during adolescence (Huttenlocher, 1979; Feinberg, 1982; Faludi and Mirnics, 2011) and the loss of parvalbumin-containing interneurons that can even lead to changes in neuronal oscillations (Perry et al., 1979; Lewis et al., 2005; Lodge et al., 2009). The functional mis-wiring can lead to the effect of the same final common pathway produced by non-specific inter-postsynaptic membrane hemi-fusions by activating non-specific sets of normal functional LINKs at the higher neuronal orders, manifesting the symptoms of schizophrenia.

During the pre-teen age when the nervous system is exposed to a large number of associative learning events, the size of the islets of functionally LINKed postsynapses increases. Pathological non-specific LINKs at this stage will have a lesser effect since the islets are small and are surrounded mostly by individual postsynapses, permitting the non-specific LINKs to induce non-specific semblances only at those individual postsynapses. The biochemical pathways that maintain the lipid membrane composition are crucial at this stage. It was reported that lipoprotein lipase is inhibited by ovarian and testicular steroids and during puberty (Bucher et al., 1997), possibly restricting the ability to re-organize the lipid composition. This is important especially in the presence of a combination of dietary lack of EFAs and the heterozygous nature of expression of proteins in the lipid synthesis and re-organization pathways. As teenagers continue to grow and associatively learn, the size of the islets of inter-postsynaptic functional LINKs increases. During this period, any membrane compositional change can increase the probability of non-specific membrane hemi-fusion between the islets, resulting in the activation of an increasing number of non-specific neurons, changes the oscillatory waveforms, leading to the internal sensations of hallucinations and cognitive defects.

## Glial Pathologies May Act Through the Final Common Pathway

Mechanisms that can induce the lateral spread of activity by non-neuronal pathologies can also lead to disease symptoms and can be explained by the present work. Changes in glial-neuronal interactions possible in oligodendroglial pathologies that are reported as white matter changes in schizophrenia (Bartzokis, 2011; Takahashi et al., 2011) may alter the relative speed of conduction in certain fibers, resulting in the simultaneous activation of a non-specific set of postsynapses inducing non-specific functional LINKs that can initiate autonomous meaningful hallucinations as explained in the earlier sections. In addition, myelin lipid changes may reflect lipid membrane compositional changes that can provoke non-specific inter-postsynaptic membrane hemi-fusion, especially in the setting of reduced expression of those proteins required for lipid transport, synthesis of essential PUFAs, and phospholipid membrane side chain replacements. In addition, extracellular matrix compositional changes (Berretta, 2012) may promote the formation of non-specific functional LINKs, generating the disease symptoms.

## Future Implications

Work toward examining the ultra-structural changes of the postsynaptic membranes is needed to test the possibility of membrane hemi-fusion as a mechanism of inter-postsynaptic functional LINK formation. Since EM examination uses sections with a thickness of 5 × 10^−8^ m, whereas the average size of a dendritic spine is nearly 4–5 × 10^−7^ m, obtaining a large number of (nearly 100) serial EM sections is necessary to examine the entire surface of a dendritic spine to visualize membrane hemi-fusion. We anticipate hemi-fusions only at very short lengths of the apposed postsynaptic membranes, necessitating a thorough examination of the entire dendritic spine’s (postsynapse’s) membrane bilayer. Since it is possible that inter-postsynaptic hemi-fusion can take place even farther away from the synaptic cleft, a large-scale re-construction of EM sections is required to visualize such hemi-fusion. The probability of finding hemi-fusion is high at regions where sensory inputs converge; for example, the hippocampus.

Adolescents with the prodrome (Yung et al., 2008; Henry et al., 2010), who have not yet presented with symptoms of schizophrenia, were shown to have a 405-fold increased risk of developing the disease compared to the general population (Cannon et al., 2008). Using a randomized double-blind placebo-controlled study, it was observed that EFA treatment prevented the development of the disease process in this group (Amminger et al., 2010). These results are explained by the role of dietary EFA in determining the postsynaptic membrane composition, in reducing abnormal membrane hemi-fusion, and in preventing conversion to psychosis. Based on the present work, how can we reverse an established disease? The traversing proteins at the non-specifically hemi-fused postsynapses need to be removed first. Identifying such proteins may lead to the development of methods to remove them from specific locations. This, combined with EFA supplementation and long-term educational programs will be required for functional rehabilitation.

## Conclusion

The present work provides the framework of a mechanism for the clinical features of schizophrenia, a disease found to be associated with a multitude of genetic and environmental factors. Non-specific hemi-fusion between postsynaptic membranes at certain neuronal orders is hypothesized to be the final common path that can induce autonomous meaningful hallucinations and cognitive defects. We expect that various other pathways that are implicated in schizophrenia, which are not mentioned in this work, can find suitable entry points into the present framework. The final path explains the implications of the involvement of both NMDA and dopamine neurotransmitters. By examining the formation of basic units of internal sensations resulting from the lateral entry of activity at the synaptic level, it was possible to attribute the diverse features of auditory hallucinations, cognitive impairment, and defects in consciousness to pathological structural changes; these changes also serve as an explanation for the beneficial effect of EFA reported from a randomized double-blinded control trial (Amminger et al., 2010). Internal sensations hypothesized as semblances induced by the lateral entry of activity at the inter-postsynaptic functional LINKs provide a testable mechanism, though testing-methods are yet to be developed. Even though the present structure-function mechanism is compatible with available experimental data, it must be considered unproven until verified against confirmatory experimental results.

## Conflict of Interest Statement

The author declares that the research was conducted in the absence of any commercial or financial relationships that could be construed as a potential conflict of interest.

## Abbreviations

AA, arachidonic acid; ACBP, acyl coenzyme A binding protein; ALA, alpha-linolenic acid; ApoB, apolipoprotein B; CA3, cornu ammonis layer 3, is a layer of neurons in the hippocampus; COMT, catechol *o*-methyl transferase; COX-1, cyclooxygenase-1; CPT-1, carnitine palmitoyl transferase-1; DAG, diacyl glycerol; DGLA, dihomo gamma-linolenic acid; DHA, docosa-hexaenoic acid; DOPA, 3,4-dihydroxyphenylalanine. It is a neurotransmitter; DPA, docosapentaenoic acid; EFA, essential fatty acids. There cannot be synthesized in the body and should be available from the diet; EPA, eicosapentaenoic acid; EPSP, excitatory postsynaptic potentials. These are potentials developed at the postsynaptic membranes when an excitatory synapse is activated; GABA, gama amino butyric acid. This is an inhibitory neurotransmitter; HDL, high density lipoprotein; IP_3_, inositol 1,4,5-trisphosphate; Islets, islets of functionally LINKed postsynaptic membranes; KMO, kynurenine 3-monooxygenase; LA, linoleic acid; LCAT, lecithin cholesterol acyl transferase; LDL, low density lipoprotein; LINK, link between the postsynapses. The words are capitalized to show its importance. Inter-postsynaptic membrane hemi-fusion is explained as a possible mechanism; LPC, lysophosphatidyl choline; LPL, lipoprotein lipase; LTP, long-term potentiation, an electro-physiological finding that has many correlations with memory; MRI, magnetic resonance imaging; NMDA, *N*-methyl d-aspartate, a chemical that specifically bind to a receptor subtype of excitatory glutamate receptors; NR1/NR2B, subunits of NMDA receptor; PC, phosphatidyl choline; PE, phosphatidyl ethanolamine; PIP_2_, phosphatidylinositol 4,5-bisphosphate; PLA2, phospholipase A2; Postsynapses, postsynaptic membranes or dendritic spines; PUFA, polyunsaturated fatty acids; SNAP, synaptosomal-associated protein; VLDL, very low density lipoprotein; ω-3, omega-3.
